# The role of aryl hydrocarbon receptor in the occurrence and development of periodontitis

**DOI:** 10.3389/fimmu.2024.1494570

**Published:** 2024-11-06

**Authors:** Lingzhi Wu, Xiting Li, Jinyu Li, Yan Wang, Canyu Yang, Chuanjiang Zhao, Li Gao

**Affiliations:** ^1^ Hospital of Stomatology, Sun Yat-sen University, Guangzhou, China; ^2^ Guangdong Provincial Key Laboratory of Stomalology, Sun Yat-sen University, Guangzhou, China; ^3^ Guanghua School of Stomatology, Sun Yat-sen University, Guangzhou, China

**Keywords:** AHR, periodontitis, tryptophan metabolism, alveolar bone homeostasis, oral microbiome-host interactions

## Abstract

Periodontitis is a condition characterized by dysbiosis of microbiota and compromised host immunological responses, resulting in the degradation of periodontal tissues. The aryl hydrocarbon receptor (AHR), a ligand-activated transcription factor, plays a crucial role in the pathogenesis of periodontitis. AHR serves as a pivotal mediator for the adverse impacts of exogenous pollutants on oral health. Research indicates elevated expression of AHR in individuals with periodontitis compared to those without the condition. However, subsequent to the identification of endogenous AHR ligands, researches have elucidated numerous significant advantageous roles associated with AHR activation in bone, immune, and epithelial cells. This review concentrates on the modulation of the AHR pathway and the intricate functions that AHR plays in periodontitis. It discusses the characteristics of AHR ligands, detailing the established physiological functions in maintaining alveolar bone equilibrium, regulating immunity, facilitating interactions between the oral microbiome and host, and providing protection to epithelial tissues, while also exploring its potential roles in systemic disorders related to periodontitis.

## 1 Introduction

Periodontitis is a persistent inflammatory condition triggered by periodontal pathogens, resulting in the degradation of soft tissues, loss of alveolar bone, and ultimately tooth loss (1, 2). The pathogenesis of periodontitis is governed by intricate signaling pathways and regulatory mechanisms. Additionally, numerous studies have linked periodontitis to various systemic diseases, establishing it as a significant risk factor for numerous chronic disorders, including Alzheimer’s disease, cardiovascular diseases, and diabetes (3).

The Aryl hydrocarbon receptor (AHR) is a transcription factor activated by ligands, both exogenous and endogenous environmental agents (4). It is expressed in various cell types, including epithelial and immune cells (5). Activation of AHR and its downstream signaling pathways have been recognized as crucial regulators of inflammation. AHR has been associated with numerous immune and inflammatory processes, including those observed in Alzheimer’s disease, rheumatoid arthritis, diabetes, cardiovascular diseases, inflammatory bowel disease, atopic dermatitis, psoriasis, and allergic reactions among other conditions (6, 7).

Numerous investigations into the AHR have elucidated a wide array of significant biological functions associated with AHR activation, including the modulation of the inflammatory microenvironment and its involvement in barrier-protective mechanisms. Over the past few years, the scholarly inquiry into AHR has progressively broadened to encompass its implications in the pathogenesis of periodontitis and the development of therapeutic interventions. Meanwhile, research observations suggest that the AHR pathway may play a significant role in systemic diseases associated with periodontitis. Understanding the involvement of AHR in disease and health is essential for comprehending variations in disease prevalence and response to treatment, and may facilitate the development of innovative therapeutic approaches. This review will specifically examine the impact of AHR on periodontal homeostasis in the context of periodontitis and related systemic disorders.

## 2 Aryl hydrocarbon receptor

### 2.1 AHR signal pathways

**Figure 1:**
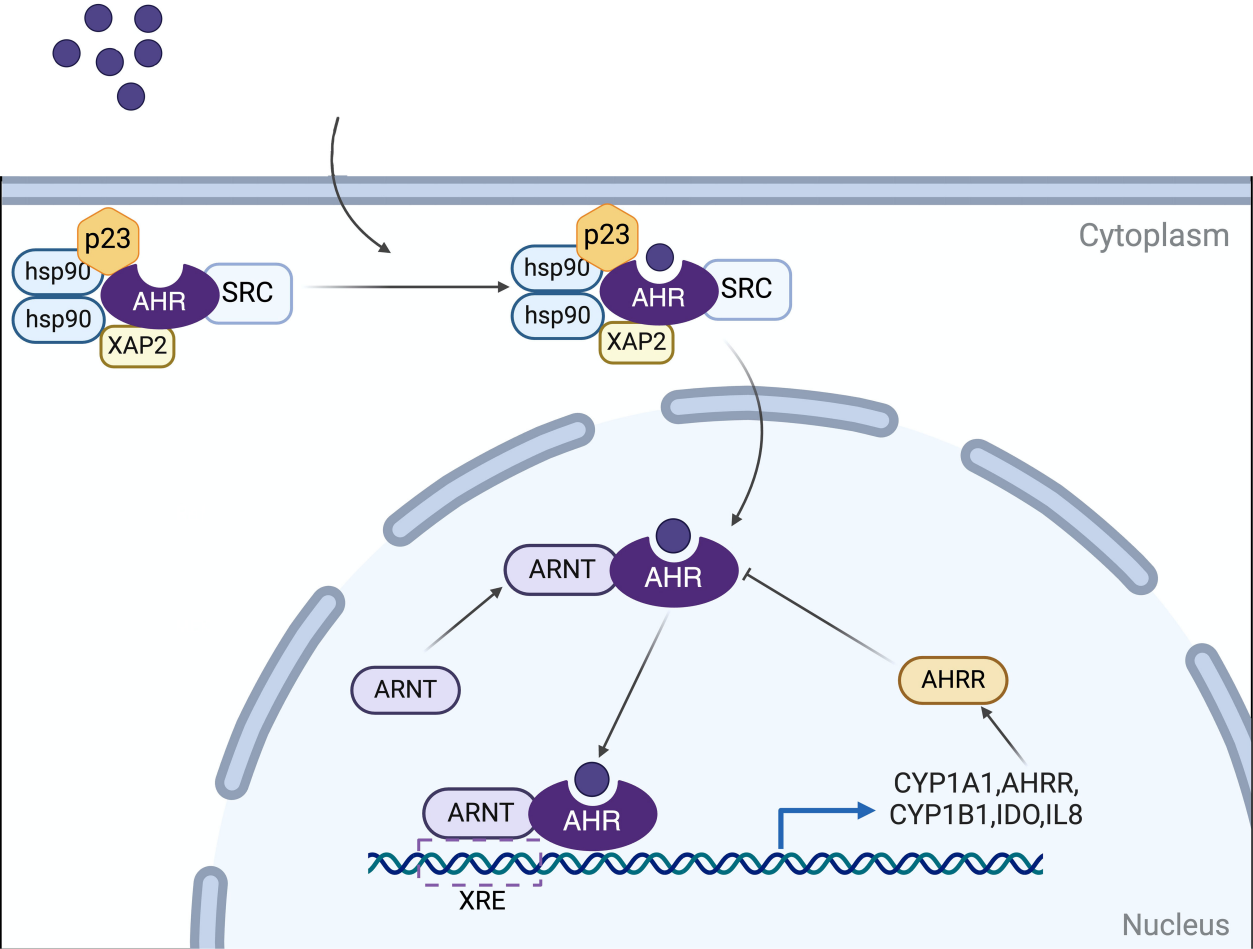
Mechanisms of AHR signaling. In its basal state, AHR associates with HSP90, p23, XAP2 (ARA9/AIP), and SRC to maintain its cytoplasmic localization. Upon ligand binding, AHR undergoes a conformational alteration and translocates into the nucleus. It dissociates from this complex and subsequently forms a heterodimer with the AHR nuclear translocation protein (ARNT). This AHR-ARNT heterodimer translocates to the nucleus, where it regulates the expression of downstream target genes by binding to xenobiotic response elements (XREs). Target genes include cytochrome P450 family 1 subfamily A member 1 (CYP1A1), CYP1B1, and AHR repressor (AHRR), among others involved in various signaling pathways. The AHRR, in turn, modulates AHR activity through a negative feedback mechanism. Created in BioRender. qwe, s. (2024) BioRender.com/z56o095.

AHR is a member of the PAS (Period [Per]Aryl hydrocarbon receptor nuclear translocator [Arnt]‐Single minded [Sim]) protein family, a highly conserved nuclear receptor (8). In its basal state, AHR associates with heat shock protein 90 (HSP90), p23, XAP2 (also known as ARA9/AIP), and the protein kinase SRC to maintain its cytoplasmic localization. Upon ligand binding, AHR undergoes a conformational alteration that exposes a nuclear localization signal (NLS), facilitating the translocation of the AHR-ligand complex into the nucleus. It has been established that HSP90 translocates to the nucleus alongside AHR (9). Nonetheless, the identification and characterization of additional cofactors that accompany AHR into the nucleus, as well as their precise roles, remain areas of incomplete understanding (5, 10, 11). After entering the nucleus, AHR forms a heterodimer with the AHR nuclear translocator protein (ARNT). This AHR-ARNT heterodimer modulates the expression of downstream target genes by binding to xenobiotic response elements (XREs), such as the cytochrome P450 family 1 subfamily A member 1 (CYP1A1), CYP1A2, and CYP1B1, as well as other signaling factors and pathways (12, 13). The pathway involving direct effects mediated through XREs is the best characterized mechanism of AHR action (**Figure 1**).

Beyond the classical pathway, the AHR regulates biological processes through non-canonical genomic and non-genomic signaling mechanisms. In addition to its direct effects mediated via XREs, AHR modulates transcriptional responses by interacting with various transcription factors and coactivators. These include the estrogen receptor (ESR), retinoic acid receptor (RAR), retinoblastoma protein (RB), Krüppel-like factor 6 (KLF6), nuclear factor erythroid 2–related factor 2 (Nrf2), musculoaponeurotic fibrosarcoma oncogene homolog (c-Maf), nuclear factor kappa-light-chain-enhancer of activated B cells (NF-κB), and members of the signal transducers and activators of transcription (STAT) family (7, 11). The AHR has been demonstrated to influence the epigenetic landscape of the cell through its regulation of noncoding RNAs, microRNAs, and mechanisms of histone acetylation and methylation (14, 15). Furthermore, AHR acts as an E3 ubiquitin ligase, facilitating the proteasomal degradation of specific target proteins (10, 16). Additionally, the activation of AHR initiates phosphorylation cascades mediated by SRC following its dissociation from the AHR chaperone complex (17).

As AHR is expressed in a wide range of cell types like epithelial cells, immune cells, endothelial cells, and neural cells, the activation of AHR has a multitude of potential consequences for various physiological functions. Studies have indicated that AHR can impact local environmental homeostasis by modulating downstream signaling pathways, potentially contributing to the pathogenesis of periodontal disease through mechanisms involving regulation of alveolar bone homeostasis, immune responses, and epithelial barrier function.

### 2.2 AHR ligands

**Figure 2:**
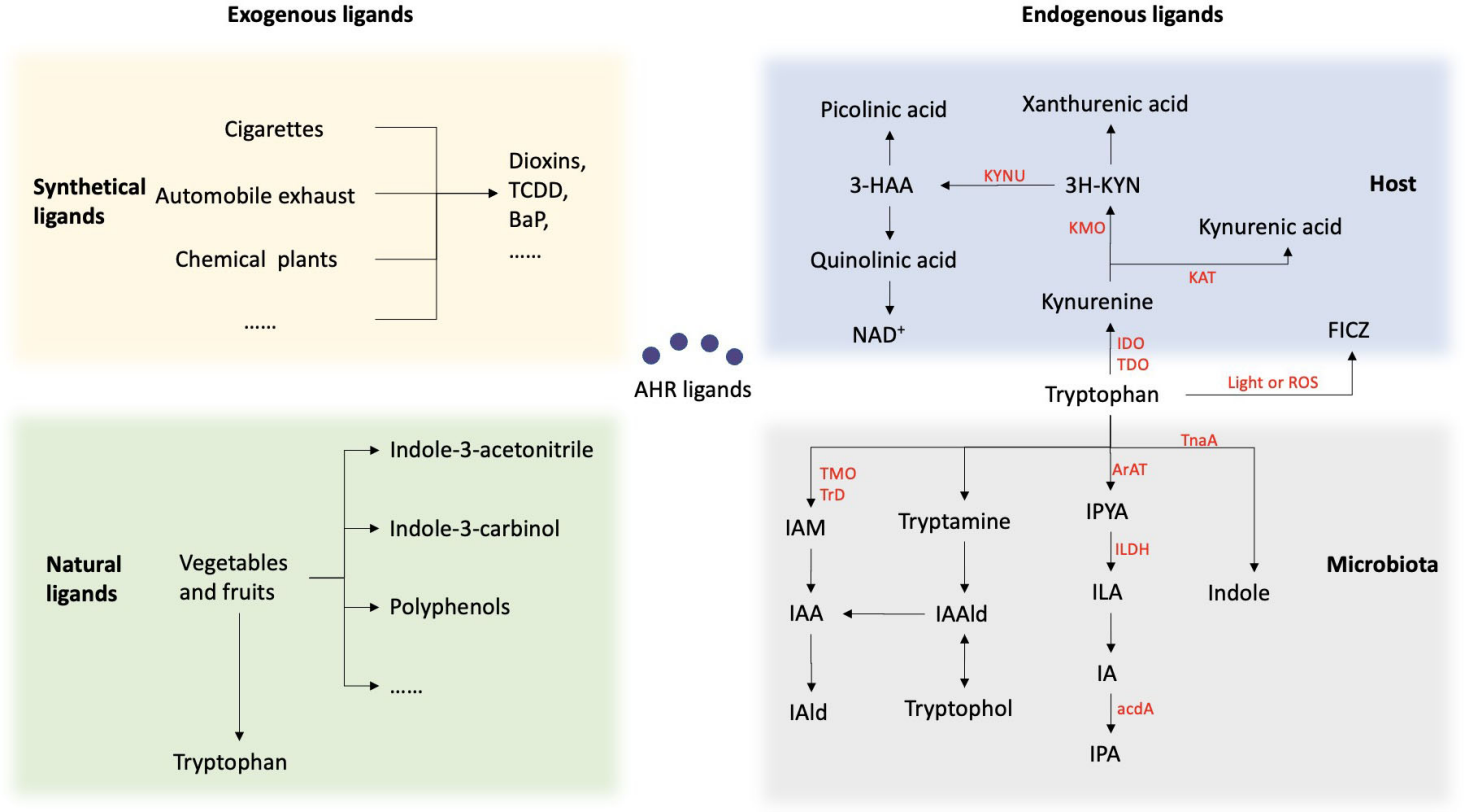
Summary of the sources of AHR ligands. AHR ligands fall into two categories: endogenous and exogenous. Exogenous ligands include synthetic aromatic hydrocarbons from pollutants and carcinogens, as well as natural compounds from vegetables and fruits. Endogenous ligands mainly come from tryptophan metabolism by the host and microbiota. Within the host, Trp is predominantly metabolized through the kynurenine (KYN) metabolic pathway. Trp is enzymatically converted by either indolamine-2, 3-dioxygenase (IDO) or tryptophan-2, 3-dioxygenase (TDO) to Kynurenine. Kynurenine undergoes conversion to kynurenic acid via the enzymatic activity of kynurenine aminotransferase (KAT), and to 3-hydroxykynurenine through the action of kynurenine 3-monooxygenase (KMO). Subsequently, it is metabolized into various products, including kynurenic acid, kynurenine, and quinolinic acid. 6-formylindolo[3,2-b]carbazole (FICZ) is produced as a result of the light or ROS in the skin. Another important source of ligand for AHR are microbial metabolites of tryptophan. Bacteria metabolize tryptophan into indole via the tnaA enzyme and can further metabolize it into indole-3-pyruvic acid (IPYA), indole-3-lactic acid (ILA), indole-3-acetaldehyde (IA), and indole-3-propionic acid (IPA). Additionally, they can convert tryptophan into tryptamine, indole-3-acetaldehyde (IAald), and tryptophol, or into indole-3-acetic acid (IAA) and indole-3-acetaldehyde (IAld).

The ligand specificity of the AHR results in varying and sometimes contradictory reactions based on the combining capacity, duration, and pathways of different ligands, highlighting the diverse effects of AHR (**Figure 2**). AHR ligands can be classified into two categories: endogenous and exogenous. Exogenous ligands, such as 2,3,7,8-tetrachlorodibenzo-p-dioxin (TCDD) and benzo(a)pyrene (BaP), are artificially synthesized aromatic hydrocarbons found in environmental pollutants and some carcinogens. The proposition of the AHR as a binding site for dioxin was introduced in the 1980s, sparking interest in toxicological investigations of synthetic aromatic hydrocarbons (18). Exogenous natural compounds, such as indole derivatives and polyphenolic substances derived from vegetables, fruits, and tropical plants, have been identified. Cruciferous vegetables belonging to the Brassicaceae family, such as cauliflower and cabbage, are particularly abundant in AHR-modulating factors that can activate the receptor in both mice and humans (12, 13).

Multiple evidence has demonstrated that the AHR is responsive to various endogenous signals originating from host metabolism and the microbiome, primarily derived from tryptophan metabolism. Tryptophan (Trp), an essential aromatic amino acid, is not synthesized within the body and is typically regarded as crucial for protein synthesis. Within the host, approximately 95% of Trp is metabolized through the kynurenine (KYN) metabolic pathway. Trp is enzymatically converted by either indolamine-2, 3-dioxygenase (IDO) or tryptophan-2, 3-dioxygenase (TDO) to Kynurenine. Kynurenine undergoes conversion to kynurenic acid via the enzymatic activity of kynurenine aminotransferase (KAT), and to 3-hydroxykynurenine through the action of kynurenine 3-monooxygenase (KMO). Subsequently, it is metabolized into various products, including kynurenic acid, kynurenine, and quinolinic acid. All of these metabolites exhibit AHR ligand activity. Furthermore, 6-formylindolo[3,2-b]carbazole (FICZ), currently the most potent AHR agonist known, is produced as a result of the photooxidation of Trp in the skin (8, 19).

Another important source of ligand for AHR are microbial metabolites of tryptophan. Bacterial Trp catabolites such as tryptamine, indole-3-acid-acetic (IAA), indole acrylic-acid (IA), indole-3-aldehyde (IAld), indole-3-propionic acid (IPA), and indole lactic acid (ILA) have been identified as ligands for AHR. Studies have demonstrated that various oral bacteria, both pathogenic and symbiotic, possess the enzymatic capability to convert Trp into indole and its derivatives. Certain subgingival plaque microorganisms, such as *Fusobacterium*, *Prevotella*, and *Porphyromonas*, possess the capacity to metabolize Trp and generate indole through Trp metabolism, resulting in halitosis and inflammation of the periodontium (20). *Lactobacilli*, another group of bacteria, possess the ability to convert Trp into IAld and ILA through the enzymatic actions of aromatic amino acid aminotransferase (ArAT), tryptophan 2-monooxygenase (TMO), and indolelactic acid dehydrogenase (ILDH) (21). Several *Bacteroides species*, as well as *Bifidobacterium* spp. have also been reported to produce ILA, IAld and IAA (22, 23).

Recent researches indicated that activation of AHR by natural ligands frequently yielded beneficial protective effects, highlighting the potential of AHR as a therapeutic target. Numerous studies have explored the therapeutic efficacy of endogenous and naturally occurring dietary AHR ligands across a range of conditions, including intestinal immune disorders, tumor immunity, neurological disorders, and autoimmune diseases. For instance, investigations have focused on the impact of supplementing with probiotics or microbial metabolites of tryptophan, such as *Lactobacillus reuteri*, *Lactobacillus bulgaricus*, indole-3-pyruvic acid, and dihydroxyquinoline, on ameliorating intestinal inflammation through AHR activation (24, 25). The advancement of AHR-targeted therapies derived from bacterial products may offer a significant alternative strategy for the management of inflammatory bowel disease (IBD). *Lactobacillus reuteri* and indole-3-aldehyde (I3A) have been shown to modulate the tumor microenvironment via AHR, thereby enhancing anti-tumor immunity and optimizing immune checkpoint function in preclinical tumor models (10, 13, 26).

However, contrasting outcomes have been observed between artificially synthesized AHR ligands and natural ligands. Traditionally, the AHR has been predominantly associated with mediating the adverse impacts of environmental toxins on the human body. This paradoxical biological effect of AHR could be attributed to the timing and dosage of ligand exposure, as well as the ligand-specific effects on AHR. Additionally, it may reflect variances in the regulation of AHR activity. The Cytochrome P450 family 1 enzymes, such as CYP1A1 and CYP1B1, are recognized for their role in negative-feedback regulation of AHR activity, with the ability to process and inactivate many AHR ligands. It is commonly believed that endogenous ligands can be efficiently metabolized by AHR-induced CYP1 enzymes, resulting in brief AHR signaling (27, 28). Conversely, artificially synthesized aromatic hydrocarbons are often poor substrates for CYP1 enzymes, leading to prolonged or sustained signaling (29, 30). These variations may contribute to differences in the response of periodontal tissue following AHR activation.

## 3 AHR and periodontitis

### 3.1 The expression of AHR ligands and AHR in periodontitis

There exist multiple AHR ligands within the oral cavity that have the potential to induce aberrant AHR expression in the context of periodontitis. Various oral pathogens have been proven to have the ability to metabolize Trp, leading to the production of indole and its derivatives (20). Elevated levels of indole have been observed in the saliva of individuals afflicted with periodontitis (31). Recent investigations have also identified the presence of kynurenine in the oral saliva of periodontitis (32). In a separate investigation, Ding et al. (33) utilized a combination of 16s rRNA sequencing and high-throughput targeted metabolomics analysis to examine the saliva microbiome and metabolome in individuals with periodontitis compared to those in good health. Their findings revealed an increase in Trp metabolism in both the dysbiotic microbiota and the patients with periodontitis. Specifically, the upregulated metabolites in patients included L-Trp, L-Kyn, and IPA. Additionally, BaP, a primary harmful component found in cigarettes, was identified as a significant exogenous ligand for the AHR (34, 35). Given the strong correlation between smoking and periodontitis, it is plausible that the AHR may exhibit heightened expression in the oral cavity of individuals who smoke and have periodontitis.

In a study conducted by J. Díaz-Zúñiga et al. (36), samples of gingival crevicular fluid, saliva, and biopsies were obtained from patients diagnosed with moderate-to-advanced chronic periodontitis, gingivitis, and healthy controls. The researchers observed significantly elevated levels of AHR and interleukin-22 (IL-22) in patients with periodontitis, which were found to be associated with osteoclast resorptive activity and disease severity. A case-control study involving the collection of tissue biopsies from volunteers diagnosed with peri-implantitis and healthy controls revealed elevated levels of gene expression of AhR and IL-6 in peri-implantitis tissues (37). Furthermore, an increased presence of CD4^+^ IL-22^+^ AHR^+^ T-lymphocytes and heightened activation of AHR were observed in periodontal lesions of infected mice compared to non-infected controls (38). However, Jing Huang et al. (39) created a periodontitis model in mice by ligating the second maxillary molar, and observed a decrease in AHR expression levels in the periodontal ligament tissue of the periodontitis group compared to the control group after 7 days. Discrepancies between clinical and animal model samples may be attributed to various factors. In clinical settings, patients with periodontitis may have higher exposure to exogenous ligands due to systemic factors like diet or smoking, whereas the ligation periodontitis model in mice typically does not involve exogenous factors. Furthermore, the duration of sampling time may also play a significant role. Clinical samples obtained from patients typically reflect a prolonged inflammatory state compared to animal experiments. Nevertheless, the conflicting outcomes observed in animal experiments remain ambiguous. This discrepancy could potentially be attributed to variations in the duration of modeling time. Mouse samples subjected to a lengthier modeling period often demonstrate elevated levels of AHR expression.

### 3.2 The role of AHR in alveolar bone homeostasis

Alveolar bone resorption is a significant pathological alteration in periodontitis, with recent research focusing on the relationship between alveolar bone resorption and bone homeostasis. Current studies have presented a cohesive understanding of the impact of alveolar bone resorption signaling on osteoblasts, indicating that activation of alveolar bone resorption can inhibit osteoblast cell differentiation. The activation of the AHR by TCDD or Kyn has been shown to hinder the differentiation of bone-marrow-derived stem cells into osteoblasts (OB), resulting in bones characterized by thin cortices, a denser matrix, and a higher trabecular fraction, ultimately leading to decreased mechanical strength (40, 41). Conversely, inhibition of AHR by resveratrol or CH223191 has been demonstrated to enhance bone mineral density and strength in murine models (42, 43). Isopsoralen (IPRN), a key bioactive compound found in Psoralea corylifolia Linn, functions as an AHR antagonist and enhances osteoblast differentiation through the AHR/ERα axis in an osteoporosis model (44). The modulation of AHR activity has varying effects on osteoblasts, with hyperactivation inhibiting and hypoactivation promoting bone formation in a dose-dependent manner. While the impact of AHR signaling on osteoblasts is well-established, the role and mechanism of AHR signaling in regulating osteoclastogenesis remains inadequately understood. The dosage and duration of agonist exposure are important variables in AHR-mediated modulation of osteoclast (OC) differentiation and function (45, 46). Low doses of the AHR ligand indoxyl-sulfate (IS) with a short exposure duration of 3 days have been shown to stimulate the differentiation of osteoclast precursor cells, whereas longer exposures of 5 days have been found to suppress osteoclast differentiation (47). Furthermore, the effects of AHR activation are influenced by both the dosage of the agonist and the cell density. BaP demonstrates pro-osteoclastogenic effects *in vitro* under conditions of low cell density, but exhibits anti-osteoclastogenic effects under conditions of high cell density (48). In summary, the AHR displays intricate regulatory mechanisms in maintaining bone homeostasis.

The cellular and histological consequences of AHR activation exhibit context-dependent variability, leading to seemingly conflicting outcomes such as the promotion or inhibition of osteogenesis. Initial investigations primarily focused on the impact of exogenous AHR agonists, specifically synthetic aromatic hydrocarbons, on alveolar bone maintenance. Researches have demonstrated that in both *in vitro* and *in vivo* settings, BaP and TCDD could enhance alveolar bone resorption through AHR pathway activation, thereby impeding osteoblast differentiation and facilitating RANKL-induced osteoclastogenesi (49–51)s. In recent years, there have been a growing body of researches examining the impact of endogenous ligands on various biological processes. Studies have demonstrated that specific endogenous ligands, such as FICZ, Kyn, and 1,25-dihydroxyvitamin D3, exhibited the ability to mitigate alveolar bone resorption in murine models of experimental periodontitis. These ligands have been found to enhance the mineralization of periodontal ligament cells (PDLCs) and inhibit osteoclast activity through the activation of the AHR via the Wnt/β-catenin and NRF2 signaling pathways (34, 39, 52, 53). On the contrary, Eisa et al. (54) discovered that Kyn enhanced RANKL-induced osteoclastogenesis *in vitro* through the activation of the AHR. The AHR’s influence on bone homeostasis is contingent upon multiple signaling pathways, such as the NF-κB pathway, Wnt/β‐catenin signaling pathways, NRF2 signaling pathways, and MAPKs signaling pathways (52, 55–58).

### 3.3 Inflammatory and immune regulation of AHR in periodontitis

Various immunocytes and inflammatory mediators are crucial in the development of inflammatory diseases, aiding in the maintenance of tissue homeostasis by establishing immunoregulatory networks to prevent harmful interactions between the host and microbiota. Nevertheless, an exaggerated immune inflammatory response in periodontitis can worsen tissue damage. It has been demonstrated that AHR activation can modulate the inflammation and immune response in periodontitis by impacting immune cells and inflammatory factors.

CD4^+^ helper T cells are essential components of the adaptive immune response within periodontal tissue. Upon activation, these cells differentiate into various subsets, including Th1, Th2, Th17, follicular helper T (Tfh) cells, and regulatory T cells (Tregs) (59). The balance between effector T cells and Tregs plays a central role in regulating periodontal inflammation. The immunomodulatory actions of AHR primarily target effector T cells and Tregs through both direct and indirect pathways. Prior research has demonstrated that activation of the AHR led to an increase in FoxP3^+^ Treg through various mechanisms, such as modulation of FoxP3 expression and direct transactivation (60). In addition, AHR activation has been shown to influence the differentiation of a subset of Th17 precursor cells into either Treg or full Th17 cell phenotypes (61, 62). Moreover, the AHR also plays a regulatory role in innate immune cells, particularly macrophages, which play a crucial role in the immune response and inflammation in periodontitis. Experimental evidence from both *in vitro* and *in vivo* studies demonstrated that Kyn activated the AHR and enhanced its binding to the promoter of NRF2, thereby promoting macrophage polarization towards anti-inflammatory M2 phenotypes (52). AHR activation has been shown to inhibit the differentiation of human monocytes into Langerhans dendritic cells (DCs) *in vitro*, while inhibition of AHR induces the differentiation of human CD34^+^ stem cell precursors into mature DCs (63). AHR activation has also been observed to regulate major histocompatibility complex class II (MHC-II) and costimulatory molecules, as well as the production of Th1 and Th17 polarizing cytokines on the surface of murine DCs (64).

The AHR is widely recognized as a transcription factor that serves as a master switch gene involved in the differentiation and function of Th22 lymphocytes, which is essential to the production and expression of IL-22, an immune-regulating factor (38). Also, AHR functions as a transcription factor that regulates the expression of numerous other inflammatory factors. The expression of AHR is markedly reduced in inflamed gingival tissue of C57BL/6 mice with experimental periodontitis and in an *in vitro* inflammation model using RAW 264.7 macrophage cells. Supplementation with an AHR agonist leads to a decrease in the production of inflammatory cytokines such as IL-6, IL-1β, and TNF-α (65). *In vitro* study also showed that AHR attenuated LPS-induced inflammation in PDLCs and reduced the expression of the inflammatory factor IL-6 through enhanced phosphorylation of STAT3 (39). The findings of another study indicated that activation of the AHR contributed to the mitigation of periodontitis and modulated the AHR/NF-κB/NLRP3 inflammasome pathway in a mouse model (53).

Multiomics analysis revealed that AHR could reduce intestinal inflammation and restore intestinal homeostasis by modulating the NF-κB signaling pathway, HIF signaling pathway, oxidative stress signaling pathway, and glycolysis (66, 67). Furthermore, the use of AHR agonists or antagonists may influence the outcomes of infectious and immune diseases, highlighting AHR as a potential target for immunotherapy (68). This implies the potential role of AHR in modulating the inflammatory milieu of the oral microenvironment in periodontitis, necessitating further experimentation to substantiate its impact and elucidate its underlying mechanism in the context of periodontal disease.

### 3.4 The role of AHR in oral microbiome-host interactions

In recent years, there has been a growing interest in the interplay between bacterial Trp metabolites and the host. Studies have demonstrated a communication pathway between the AHR and microbial communities, potentially influencing host homeostasis. The oral microbiota has the capability to generate indole and its derivatives through bacterial Trp metabolism, leading to the activation of the AHR signaling pathway and the regulation of oral homeostasis. Yan et al. (69) identified a strong correlation between Trp metabolism and AHR activation in chronic obstructive pulmonary disease (COPD), with *Lactobacillus* species (specifically *Lactobacillus salivarius* and *Lactobacillus oris*) playing a predominant role, followed by *Streptococcus* and *Neisseria flavus*. The gut microbiota, known as the primary metabolic site for amino acids, has been extensively researched for its ability to produce AHR ligands like IAA, ILA, and IAld through Trp metabolism. Multiomics analysis demonstrated a positive correlation between microbial Trp metabolism and the host’s AHR/IL-22 signaling pathway, while also revealing significant negative correlations with host epithelial cell apoptosis, IL-1 signaling, and oxidative stress modules. Nevertheless, there is a notable absence of similar multiomics studies focusing on the oral environment. Like the intestinal tract environment, *Lactobacillus*, a crucial symbiotic bacterial group in the oral cavity, may possess the capability to metabolize AHR ligands, thereby potentially mitigating periodontal tissue inflammation and enhancing overall periodontal health.

Alternatively, the AHR in the host has the potential to impact the population of microbiota and facilitate a state of equilibrium between the host and microbes. Renga et al. (70, 71) discovered that in mice experiencing colitis, 3-IAld has the ability to alter the composition and function of intestinal microbiota, favoring the growth of sugar-fermenting bacteria and the localized production of short chain fatty acids (SCFA) through the activation of the host AHR/IL-22-dependent pathway. The AHR/IL-22 pathway regulates the composition and function of the microbiome through various direct and indirect mechanisms. Upon activation of the AHR, intestinal epithelial cells (IECs) increase expression of proliferative and anti-apoptotic pathways, secretion of antimicrobial proteins (AMPs), as well as production of mucin and fucosylation (72). The AHR/IL-22-induced proliferative response and AMP secretion by IECs may eliminate specific bacterial clades, serving as a negative selective force on the microbiota (73). On the contrary, the regulation of mucin production and fucosylation by AHR/IL-22 leads to the selection of commensal organisms that enhance colonization resistance against Clostridioides difficile and promote the growth of microbes capable of metabolizing host-derived sugars, thereby exerting a beneficial selective pressure (74). Both *in vivo* and *in vitro* studies have demonstrated that IAA derived from the airway microbiome influence the composition of the airway microbiota by promoting the proliferation of *Lactobacillus* as an AHR ligand, highlighting the significance of targeting AHR in host-microbial interactions (69). Additional research is warranted to investigate the function and mechanism of the AHR in host-microbial interactions in periodontitis.

### 3.5 The potential protective effect of AHR in gingival epithelial barrier

The gingival epithelium serves as the initial protective barrier of periodontal tissue and acts as the primary defense against bacterial assaults (75). While research on the role of AHR in the gingival epithelial barrier is limited, numerous studies have focused on its function in other epithelial barriers, including the intestinal mucosa, pulmonary epithelium, and skin epithelium (76, 77).The identification of the AHR as a crucial regulator of homeostasis in barrier tissues such as the skin, lungs, and intestines has been well-established.

The activation of the AHR pathway plays important role in maintaining mucosal barrier function through various mechanisms. It shows effectiveness in strengthening the intestinal mucosal barrier by modulating the expression and localization of tight junction (TJ) and adhesive junction (AJ) proteins (5, 78). Both *in vitro* and *in vivo* experiments have indicated that the expression levels of key TJ and AJ proteins, such as zonula occludens protein 1 (ZO-1), claudin-1, occludin, and E-cadherin, were reduced in experimental models of inflammatory bowel disease but significantly increased following treatment with AHR agonists FICZ or IAld (79). Additionally, the activation of the AHR was found to preserve the integrity of the epithelium and mitigate the disruption of cellular trans-epithelial electrical resistance (TEER) within the intestinal epithelial barrier in cases of inflammatory bowel disease. AHR also regulated cell proliferation and differentiation, as well as decreased the heightened apoptosis of epithelial cells induced by various diseases (19, 78, 80).

In order to further decipher the mechanisms, recent studies conducted in mice and intestinal epithelial cells have elucidated that the activation of the AHR could suppress the activation of myosin light chain kinase (MLCK) and phosphorylated myosin light chain (pMLC) signaling pathway, thereby alleviating intestinal mucosal barrier dysfunction (79, 81). Another study discovered that certain indole derivatives safeguarded the epithelial barrier from heightened gut permeability by preserving the integrity of the AJ complex and its associated actin regulatory proteins, such as myosin IIA and ezrin, and their effects were dependent on the AHR (19). In addition, AHR has been found to promote the expression of the IL-22 and improve the destruction of the epithelial barrier via the AHR/IL-22 signaling pathway (70). Furthermore, the AHR is activated by a Trp microbial metabolite to bolster gut barrier integrity via the Nrf2 pathway (78, 82).

**Figure 3:**
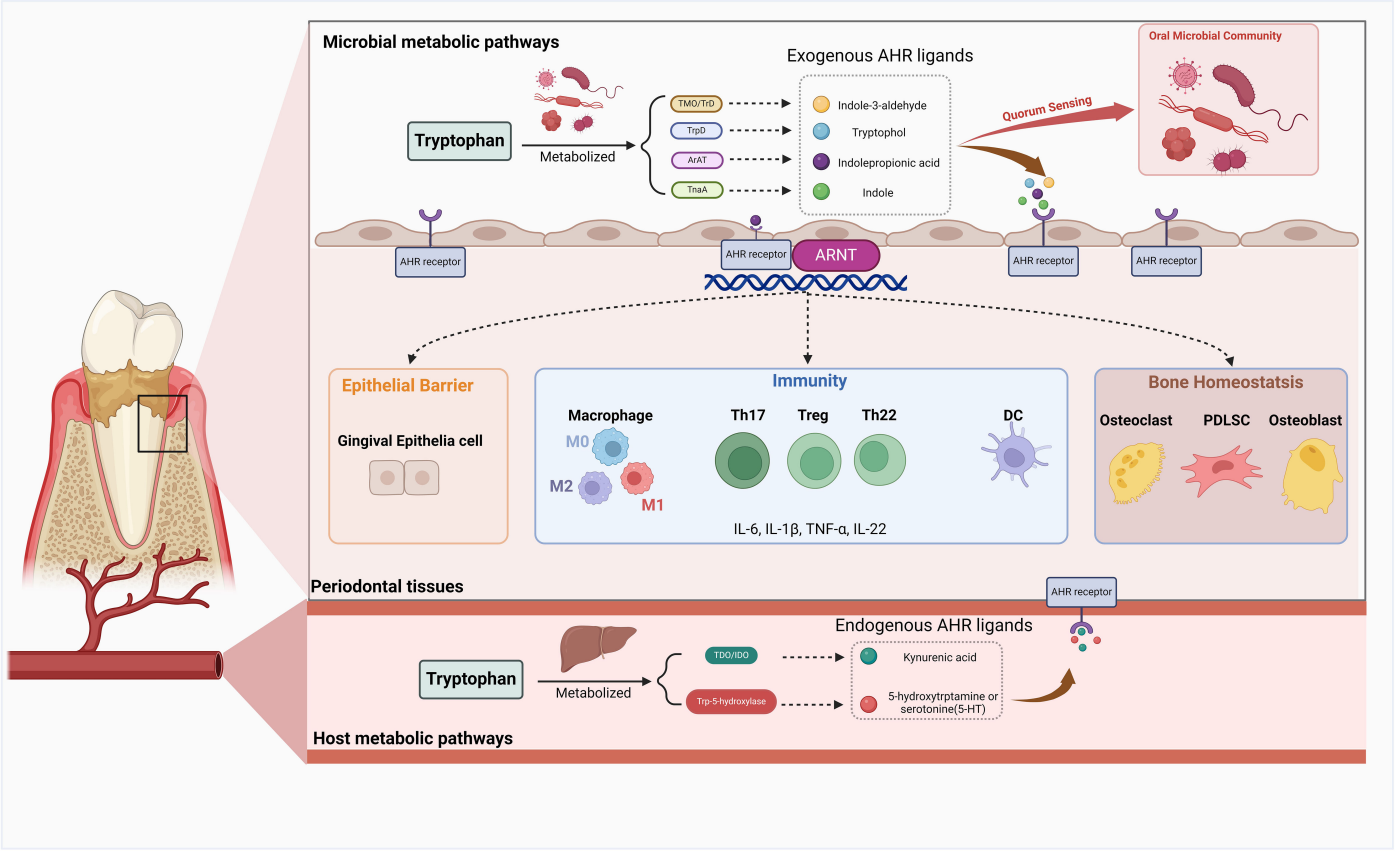
The role of AHR in the periodontal tissue. Upon activation by exogenous or endogenous ligands, the Aryl Hydrocarbon Receptor (AHR) exerts a multitude of functions within periodontal tissue. AHR demonstrates intricate regulatory mechanisms in maintaining bone homeostasis by modulating the biological processes of osteoblasts, osteoclasts, and periodontal ligament cells (PDLCs). Activation of AHR can influence inflammation and immune responses in periodontal tissue by affecting the equilibrium between effector T cells and regulatory T cells (Tregs), macrophage polarization, dendritic cell (DC) differentiation, and the production of various inflammatory mediators. AHR activation in the host also has the potential to promote a state of equilibrium between the host and microbial communities and exert a protective effect on the gingival epithelial barrier. Created in BioRender. Feng, B. (2024) BioRender.com/v13j704.

Although limited studies have been conducted, it is plausible that AHR may confer a comparable protective influence on the gingival epithelial barrier, which deserves further exploration (**Figure 3**).

## 4 AHR in the relationship between periodontitis and systemic diseases

**Figure 4:**
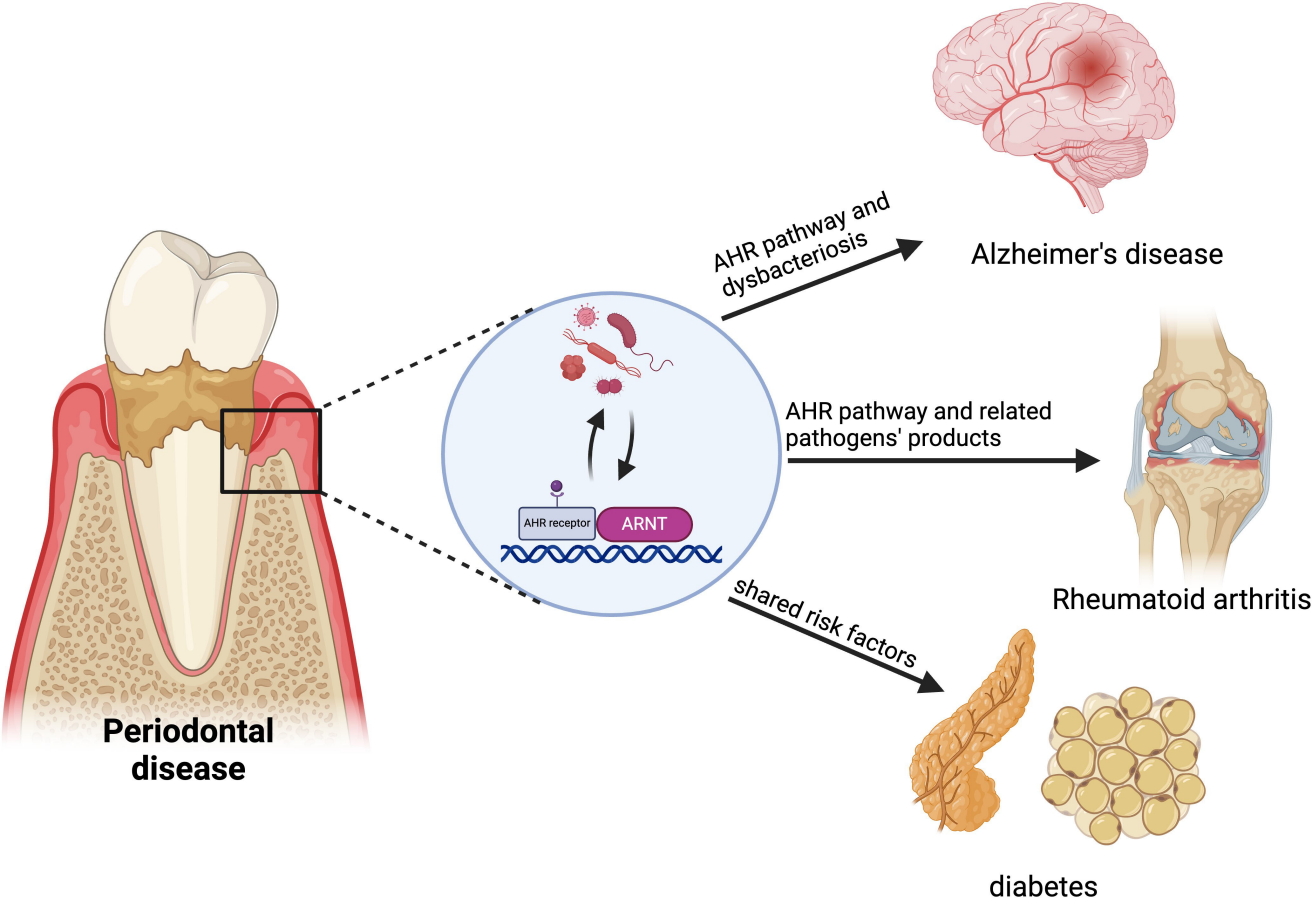
AHR in the relationship between periodontitis and systemic diseases. AHR links periodontitis to systemic diseases like Alzheimer’s, rheumatoid arthritis (RA) and diabetes. Periodontitis may increase Alzheimer’s risk by disrupting brain vascular homeostasis and the blood-brain barrier through AHR signaling. Pathogens from periodontitis affect immune balance, potentially accelerating RA progression via abnormal AHR activation. Synthetic aromatic hydrocarbons represent a common risk factor for both periodontitis and diabetes. Created in BioRender. Feng, B. (2024) BioRender.com/v59o971.

Periodontitis is widely acknowledged to have strong associations with various systemic diseases. Emerging evidence demonstrates that the AHR may be implicated in the pathogenesis of several inflammatory and immunological conditions. The alteration of AHR expression induced by periodontitis may have significant implications in the pathophysiology of systemic disorders linked to periodontitis, including Alzheimer’s disease, diabetes, and rheumatoid arthritis (**Figure 4**).

### 4.1 Alzheimer’s disease

Alzheimer’s disease (AD) is a neurodegenerative condition characterized by the accumulation of beta-amyloid (Aβ) plaques in the brain, resulting in chronic neuroinflammation (83). Periodontitis has been identified as a potential risk factor for AD and is associated with cognitive impairment (84–86). Administration of nanoparticles to restore gingival macrophage function has been shown to decrease inflammation in periodontal tissue, reduce the number of astrocytes in the hippocampus, and prevent cognitive decline (87).

Numerous studies have indicated that L-Trp metabolites and SCFAs are the main messengers in the microbiota-brain axis (83). The AHR serves as the primary target for Trp metabolites within the cerebral microvasculature. Dysbiosis of gut microbiota impairs the integrity of blood-brain barrier via the activation of AhR signaling and thus aggravates AD pathology. Wu et al. (88) observed significant variations in gut microbial metabolites between healthy individuals and those with mild cognitive impairment (MCI) or AD, with a notable increase in IPA levels. The conversion of microbiota-generated indole molecules into indoxyl sulfate (IS) in the human liver has been shown to have deleterious effects on vascular homeostasis and AD pathology (89). Teruya et al (90) found a significant elevation in the levels of Kyn and IS, both of which are activating ligands of the AHR, in the blood samples of dementia patients. An aberrant activation of AHR signaling disrupts vascular homeostasis in the brain and compromises the integrity of the blood-brain barrier. The dysbacteriosis in periodontitis may influence the development of AD through AHR signaling (91). Certain key subgingival periodontal pathogens, such as *Porphyromonas gingivalis*, have been detected in the brains of individuals with AD. Infection of *Porphyromonas gingivalis* in mice has been shown to result in brain colonization and an elevation of amyloid plaque levels (92, 93). Given the pathogens’ ability to metabolize Trp to generate AHR agonists, it is conceivable that the AHR may serve as a target for periodontal pathogens in facilitating the advancement of AD.

On the contrary, some researches indicated that specific medications have the potential to modulate microbial dysbiosis via the AHR, thereby facilitating the degradation of Aβ and mitigating neuroinflammation. This suggests that AHR could serve as a viable therapeutic target for addressing AD (24, 94). Discrepancies in physiological outcomes may be attributed to variations in dosage, duration of treatment, and the specific ligands involved in AHR activation. The strategic activation of the AHR pathway may represent a shared therapeutic approach for both periodontitis and AD. The ongoing investigation in this area shows promise and warrants further exploration.

### 4.2 Rheumatoid arthritis

Findings from clinical and epidemiological studies indicate a potential association between rheumatoid arthritis (RA) and an elevated susceptibility to periodontitis and tooth loss (95). Furthermore, there is evidence supporting the notion that periodontitis may play a contributory role in triggering and perpetuating the autoimmune inflammatory response characteristic of RA (96). A study has highlighted the disruptive impact of periodontitis-related pathogens, such as *Porphyromonas gingivalis*, and their byproducts on the host’s immune equilibrium, with the AHR potentially serving as a specific mechanism linking these processes (97).

A latest research highlighted the significant involvement of AHR in the pathogenesis and therapeutic strategies for RA (24, 98). Environmental pollutants and cigarette smoke, known risk factors for RA, contain AHR agonists (99). Given smoking is also an important risk factor for periodontitis, it is postulated that the exogenous activation of AHR may play an important role in the onset and progression of both conditions. Several studies have investigated the impact of exogenous ligand TCDD on RA. Murine models of RA have illustrated that AHR activation by TCDD contributed to RA disease progression, disease severity, bone degradation, and osteoclasts differentiation in inflamed joints. Additionally, analysis of clinical samples has revealed that AHR expression was approximately two times greater in RA patients compared to control subjects (99).

However, certain endogenous metabolites and natural ligands have been shown to relieve the severity of rheumatoid arthritis by activating the AHR (100). Sinomenine (SIN) is a potent immunosuppressive and anti-inflammatory agent utilized in the treatment of RA. Both *in vivo* and *in vitro* studies have demonstrated that SIN can mitigate inflammation and maintain the balance between Th17 and Treg cells in collagen-induced arthritis rats. This effect is primarily mediated through the elevation of microbial Trp metabolites such as IA, IPA, and IAA, as well as the activation of AHR (101). Endogenous ligands have been shown to have similar effects in periodontitis. In a study by Rosser EC et al (24), supplementation with SCFAs such as butyrate was found to decrease arthritis severity by elevating levels of the serotonin-derived metabolite 5-Hydroxyindole-3-acetic acid (5-HIAA), which in turn activated the AHR in IL-10-producing regulatory B cells (Bregs).

Hence, the potential linkage between periodontitis and RA may be attributed to the aberrant activation of AHR signaling pathways.

### 4.3 Diabetes

Diabetes is distinguished by persistent subclinical inflammation, akin to that observed in periodontitis. Extensive research has explored the reciprocal influences between these two conditions (102). Diabetes can precipitate an exaggerated inflammatory reaction to periodontal microbiota, thereby hastening periodontal deterioration. Meanwhile, periodontitis can detrimentally impact glycemic regulation in individuals with diabetes, and lead to the development of diabetic complications.

Studies on the correlation between diabetes and AHR primarily examined the toxicological impact of exogenous substances like TCDD. Increasing evidence indicates that AHR signaling activated by toxins disrupts fat metabolism, glucose regulation, and insulin release, resulting in metabolic dysfunction (103, 104). This disruption may be facilitated through mechanisms such as gluconeogenesis, the hypoxia-inducible factor (HIF) pathway, oxidative stress, and inflammation (105). Toxicological investigations of TCDD in periodontitis have demonstrated its capacity to induce alveolar bone resorption and expedite the progression of periodontitis, indicating a shared risk factor for both periodontitis and diabetes. However, there is a paucity of research focusing on the impact of endogenous metabolites on the pathophysiology of diabetes, necessitating additional elucidation.

## 5 Conclusions and future perspectives

This review provides a comprehensive overview of the role and potential mechanisms of the AHR and its pathways in the initiation and advancement of periodontitis, as well as its impact on periodontitis and associated systemic diseases. The AHR pathway plays a significant and intricate role in the oral microenvironment. Exogenously synthesized AHR agonists may induce toxic effects and pose a risk factor for periodontitis through prolonged activation of the AHR pathway. Regarding endogenous AHR ligands, researches have demonstrated that activation of the AHR pathway by endogenous molecules or microbial metabolism plays a role in regulating alveolar bone homeostasis and enhancing the oral immune system. Studies conducted in various microenvironments, including the gut, have indicated that AHR can bolster the epithelial barrier and modulate the balance of flora to optimize the microenvironment. This suggests that AHR may have the capacity to fortify the gingival epithelial barrier and regulate oral microbiota. The intricate biology of the AHR and its implications underscore the importance of thoroughly investigating AHR ligand-receptor interactions, mechanisms, and downstream biological processes in the context of periodontitis. Future research efforts should prioritize examining the synergistic and inhibitory relationship between AHR pathway activation and the progression of periodontitis in order to achieve a more nuanced understanding of this complex interplay.
